# Simple Sequence Repeat-Based Genetic Diversity Analysis of Alfalfa Varieties

**DOI:** 10.3390/ijms26115246

**Published:** 2025-05-29

**Authors:** Jie Wang, Xiaoli Wei, Changying Guo, Chengti Xu, Yuanyuan Zhao, Xiaojian Pu, Wei Wang

**Affiliations:** 1 Academy of Animal Science and Veterinary Medicine, Qinghai University, Xining 810016, China; wangjie08142023@163.com (J.W.); 18894499178@163.com (X.W.); gchangying2021@163.com (C.G.); xchti@163.com (C.X.); 18893147262@163.com (Y.Z.); 2 Key Laboratory of Northwest Cultivated Land Conservation and Marginal Land Improvement Enterprises, Ministry of Agriculture and Rural Affairs, Delingha 817000, China

**Keywords:** alfalfa, SSR, forage breeding

## Abstract

Alfalfa, as a high-quality forage resource, has high nutritional value. Due to the high phenotypic similarity among its varieties and the susceptibility to environmental influences, challenges are encountered in variety identification and breeding. In this study, 23 simple sequence repeat (SSR) markers were screened to distinguish 49 alfalfa varieties, among which 21 SSR markers showed polymorphic fragments. The results indicated that these 21 markers were highly polymorphic, with an average of 5.91 alleles per SSR marker locus and an average polymorphic information content (PIC) of 0.66, suggesting a strong discriminatory efficiency. The results of a population genetic diversity analysis showed that there was a relatively high level of genetic diversity among the tested materials. The analysis of molecular variance (AMOVA) results indicated that the genetic variation within the population of the 49 alfalfa germplasm samples was the main source of the total variation. The results of genetic distance and genetic identity analyses showed that the genetic relationship between population 1 and population 4 was the most distant, while the relationship between population 2 and population 3 was the closest. The cluster analysis results showed that samples S16 and S55 formed a separate branch; that is, there were two main genetic subgroups. These results confirm that SSR markers are effective tools for genetic characterization and precise discrimination of alfalfa varieties and have important application values in breeding, variety registration, and germplasm resource conservation.

## 1. Introduction

Alfalfa (*Medicago* spp.) belongs to the legume family, primarily consisting of herbaceous plants with a few shrubs and subshrubs, and can be either annual or perennial. It is the earliest cultivated and most widely distributed legume forage globally, renowned as the ‘King of Forages’ [1]. Alfalfa is characterized by high yield, excellent quality, and strong adaptability, playing a significant role in the development of animal husbandry and the protection of the ecological environment [2,3]. Alfalfa is cultivated for hay and silage production, and it is also utilized for grazing or dehydrated to produce alfalfa powder or pellets [4]. This legume is rich in conventional nutrients such as proteins, vitamins, amino acids, and macroelements. It also contains various chemical compounds, including saponins, flavonoids, and alkaloids [5,6]. Alfalfa serves as a vital source of high-quality forage in the northwest region and plays a crucial role in the development of the livestock industry [7]. Furthermore, its well-developed root system enhances vegetation interception and soil infiltration, thereby improving water retention and soil stabilization. The symbiotic rhizobia present in its roots can enhance soil fertility [8,9] and increase the organic matter and humus content [10], making alfalfa a commonly used plant for pollution remediation and soil condition improvement [11]. Despite the long history of alfalfa cultivation in China, the intensive and industrial development of this crop has progressed slowly. Consequently, the domestic supply fails to meet the demands of the livestock industry, resulting in a significant reliance on imports of high-quality alfalfa from abroad. Between 2012 and 2020, the import volume of alfalfa hay in China increased from 440,000 tons to 1.36 million tons [12], while the average self-sufficiency rate for high-quality alfalfa was only 64% [13]. Therefore, exploring ways to enhance the production capacity of alfalfa in China is crucial for the sustainable development of the livestock industry. The primary challenges include a scarcity of local variety resources, outdated breeding techniques, and insufficient cultivation practices. Currently, research by Chinese scholars primarily focuses on variety introduction, cultivation practices, stress resistance evaluation, and the mining of stress resistance genes, resulting in significant advancements in the utilization of alfalfa germplasm resources and understanding of stress resistance mechanisms. However, there is relatively less research on genetic diversity and breeding [14,15]. Additionally, extensive studies on the introduction of alfalfa have enriched alfalfa germplasm resources of China; however, numerous challenges remain in production across different regions, particularly with the varying performance and nutritional quality of introduced varieties in different areas.

Molecular marker technology, which is based on individual nucleotide sequence variations, analyzes genetic diversity at the DNA level [16]. Unlike morphological markers, molecular markers are not influenced by environmental factors or by gene dominance and recessiveness. They can cover the entire genome with a large number of markers exhibiting high polymorphism [17]. The main molecular marker technologies include Restriction Fragment Length Polymorphism (RFLP) [18], Random Amplified Polymorphic DNA (RAPD) [19], Inter-Simple Sequence Repeat Polymorphism (ISSR) [20], Single-Nucleotide Polymorphism (SNP) [21], Amplified Fragment Length Polymorphism (AFLP) [22], and simple sequence repeats (SSRs) [23]. Microsatellite markers, also known as short tandem repeats or simple sequence repeats, are simple repetitive sequences that are uniformly distributed in the genomes of eukaryotes. They consist of tandemly repeated segments of 2 to 6 nucleotides. The number of repeat units in microsatellites shows high variability, manifesting as integer-multiple variations in the number of microsatellite units or potential differences in the sequences of the repeat units, thus creating polymorphism at the loci [24].

SSR marker loci are randomly distributed on DNA, adhere to Mendelian inheritance laws, and exhibit strong stability and reproducibility, which makes them widely applicable [25]. SSR markers have been extensively studied in various aspects of alfalfa. Sakiroğlu et al. utilized SSR marker technology to infer the population structure and genetic diversity of a wide range of wild diploid alfalfa germplasm resources [26]. Azzam et al. applied SSR markers to investigate the relationship between saponin concentration and the enhancement of resistance to damping-off in alfalfa seedlings [27]. Min et al. developed miRNA-SSR markers for alfalfa using SSR marker technology and assessed their interspecies transferability among six legume species [28]. These studies demonstrated that SSR markers had been employed in diverse areas of alfalfa research and are likely to be applied in additional research directions in the future. Fluorescence capillary electrophoresis (FCE) is an analytical method that combines fluorescence detection technology with capillary electrophoresis [29]. Compared with the traditional polyacrylamide gel electrophoresis, the SSR fluorescence-labeled capillary electrophoresis detection technology has the advantages of high efficiency, accuracy, and automation [30]. It overcomes the deficiencies of polyacrylamide gel electrophoresis. It can not only accurately read the size of the product fragments but also has a lower cost, showing great application value. Its identification results are not easily affected by time and the environment, indicating that fluorescence capillary electrophoresis can improve the accuracy of the results in an SSR analysis [29,31]. At present, it is applied in the fields of life science [32], medicine [30], environmental science [33], etc.

Research on the genetic diversity of alfalfa seed germplasm resources using molecular markers is currently limited [34]. Therefore, to accelerate the development of the alfalfa industry in China and meet the demands of the livestock sector, it is essential to fully exploit both domestic and international variety resources. This includes analyzing the genetic diversity of alfalfa, identifying the genetic relationships among germplasm resources, and selecting high-yield and high-quality alfalfa varieties. This paper employs SSR molecular marker analysis of 49 alfalfa germplasm resources from various countries, further elucidating the genetic diversity of these resources and identifying the genetic relationships among the germplasm resources, which holds significant implications for future breeding efforts.

## 2. Results

### 2.1. SSR Primer Screening

Initially, a total of 102 pairs of primers were screened using eight samples (S8, S13, S16, S25, S34, S37, S47, and S50). Eventually, 23 pairs of polymorphic primers were selected (Table 1).

### 2.2. Genetic Diversity Analysis

From a total of 102 pairs of SSR marker primers, 23 pairs exhibiting polymorphism were identified, allowing for the study of genetic diversity among 49 alfalfa varieties (Table 2). The results indicated that these 23 pairs of primers amplified a cumulative total of 136 alleles, of which 129 were polymorphic. The number of alleles produced by each primer pair varied, ranging from 3 (for AW282, BF123, MT1E04, and MTIC249) to 16 (for BE112), with an average of 5.91 alleles per pair. Similarly, the number of polymorphic alleles generated by each pair ranged from 2 (for AW282 and MTIC249) to 16 (for BE112), with an average of 5.61 polymorphic alleles. The PIC varied from 0.347 to 0.804, yielding an average value of 0.66. When the PIC value > 0.5, it indicates that the marker has high polymorphism; when 0.25 ≤ PIC≤ 0.5, it shows that the marker has moderate polymorphism; and when PIC < 0.25, it means that the marker has low polymorphism. In this study, primers with high PIC values accounted for 86.95%, indicating that these SSR loci can explain genotypic differences at the molecular level and exhibit rich genetic diversity.

### 2.3. Genetic Diversities of the Population

Based on the PCR amplification banding patterns of 23 SSR molecular markers, the 49 alfalfa germplasms were classified into four populations. The first category includes eight germplasms: S11, S12, S13, S14, S15, S16, S17, and S18. The second category comprises 25 germplasms, specifically S1, S2, S3, S21, S22, S23, S25, S26, S27, S28, S29, S30, S31, S32, S33, S34, S35, S36, S37, S38, S39, S40, S41, S42, and S43. The third category consists of 12 germplasms: S4, S44, S45, S46, S47, S48, S49, S50, S52, S53, S54, and S55. The fourth category includes four germplasms: S5, S7, S8, and S56.

Genetic diversity analysis of the four populations of the tested materials was conducted based on SSR markers, and the results are shown in Table 3. The range of observed alleles (Na) in the four populations was from 0.941 to 1.581, with an average of 1.2445; the range of effective alleles (Ne) was from 1.25 to 1.32, with an average of 1.2925; the range of the genetic diversity index (H) was from 0.147 to 0.192, with an average of 0.1768; the range of the Shannon information index (I) was from 0.216 to 0.303, with an average of 0.2695; and the range of the unbiased diversity index (UH) was from 0.196 to 0.209, with an average of 0.202. These results indicate that there is a relatively high level of genetic diversity among the populations of the tested materials.

### 2.4. Analysis of Molecular Variance (AMOVA)

The results of the AMOVA are shown in Table 4. They indicate that 2% of the genetic variation exists among populations, while 98% exists within populations. There is significant variation among the populations of the germplasm materials (*p* < 0.001). This suggests that the genetic variation within the populations of the 49 alfalfa germplasm samples is the main source of the overall variation.

### 2.5. Analysis of Genetic Distance and Genetic Identity

Based on Nei’s index, the genetic distances and genetic identities among the populations of the tested materials were obtained (Table 5). The genetic identity among the four populations ranged from 0.952 to 0.980, with an average of 0.9625, and the genetic distance ranged from 0.020 to 0.052, with an average of 0.0297. Among the four populations, the genetic relationship between Population 1 (pop1) and Population 4 (pop4) was the most distant (Gs = 0.952, Gd = 0.049), while the relationship between Population 2 (pop2) and Population 3 (pop3) was the closest (Gs = 0.980, Gd = 0.020).

### 2.6. Principal Coordinate Analysis

**Figure 1:**
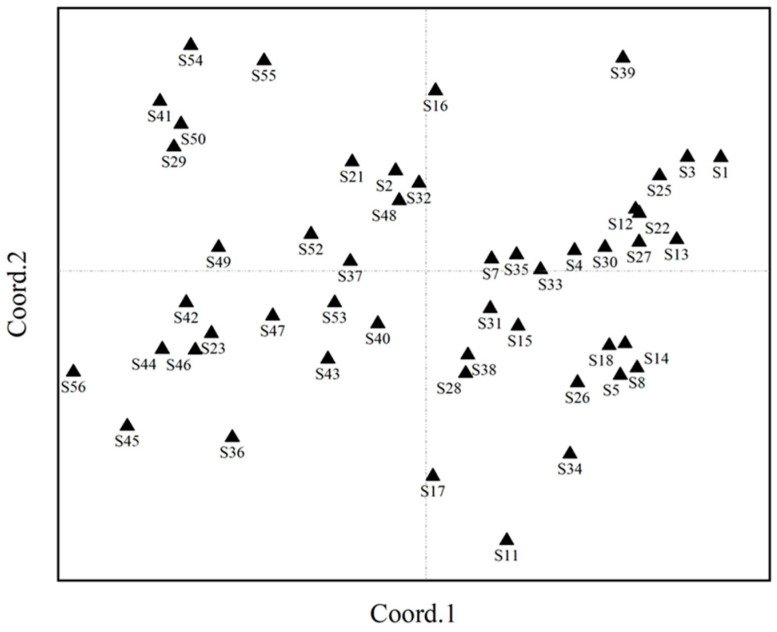
Principal coordinate analysis of SSR markers.

A principal coordinate analysis (PCoA) effectively illustrates the variations between samples by intuitively comparing the straight-line distances among them on the coordinate axes. A smaller straight-line distance between samples indicates lesser differences, while a greater distance signifies more substantial differences. The resulting PCoA diagram bears resemblance to the clustering results obtained from SSR molecular markers (Figure 1). It is noteworthy that the 49 alfalfa varieties are relatively dispersed, indicating a rich diversity of genetic resources.

### 2.7. Cluster Analysis

**Figure 2:**
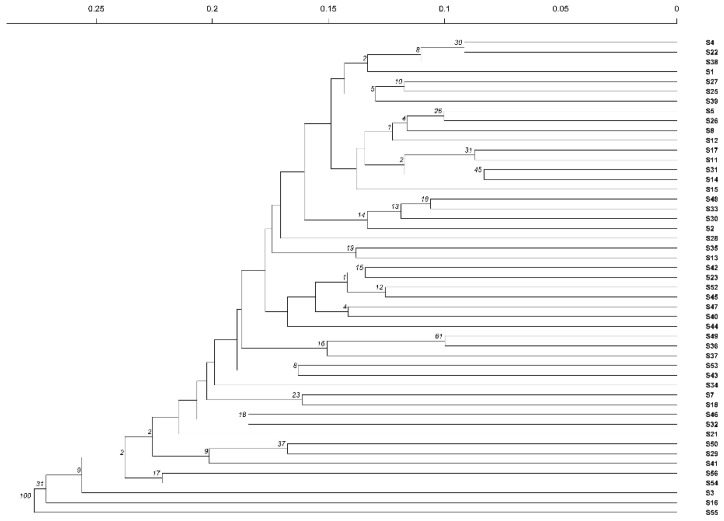
Cluster analysis of SSR markers.

A cluster analysis using the Unweighted Pair-Group Method with Arithmetic Mean (UPGMA) method was conducted on 49 samples, previously grouped in four populations of the tested materials, based on genetic distance and genetic identity. At a genetic distance of 0.18, the materials were divided into two major groups (excluding S16 and S55). The first group comprised 17 materials, namely S3, S54, S56, S41, S29, S50, S21, S32, S46, S18, S7, S34, S43, S53, S37, S36, and S49, with 1 from pop1, 8 from pop2, 6 from pop3, and 2 from pop4 (Figure 2). The second group consisted of 30 materials, which were further divided into two subgroups. The first subgroup included 14 materials, namely S44, S40, S47, S45, S52, S23, S42, S13, S35, S28, S2, S30, S33, and S48, with 1 from pop1, 8 from pop2, and 5 from pop3 (Figure 2). The second subgroup comprises 16 materials, namely S15, S14, S31, S11, S17, S12, S8, S26, S5, S39, S25, S27, S1, S38, S22, and S4. Among these, five belong to pop1, eight to pop2, one to pop3, and two to pop4 (Figure 2).

### 2.8. Construction of DNA Fingerprint Map

**Figure 3:**
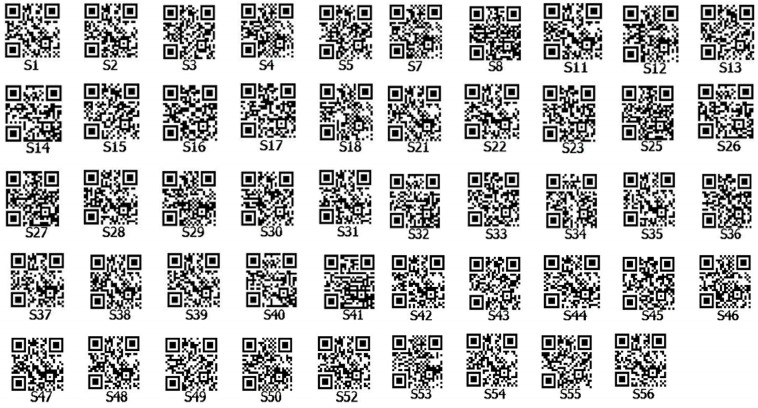
Fingerprint profiles of 49 alfalfa varieties. **Note:** According to the size of amplified fragments in each sample, an original data matrix was constructed (denoted as “1” for the presence of bands and “0” for the absence of bands). The amplification results (1/0) were concatenated into a string of binary codes, which serve as the fingerprint codes for varieties to distinguish different alfalfa cultivars.

DNA fingerprints can transform the variations in DNA levels among different samples into QR codes, thereby presenting sample-related information more intuitively. Following the amplification of 49 alfalfa varieties using 23 pairs of primers, the number of amplified fragments and their sizes were recorded. A fingerprint code was established for the tested alfalfa, and this variety information, along with the fingerprint code, was utilized to generate a fingerprint QR code (Figure 3).

## 3. Discussion

Alfalfa is characterized by its extensive genetic variability, which enables it to adapt to a wide range of environments, from extremely hot to extremely cold [35]. SSRs, one of the most commonly used genetic markers, play a significant role in plant genetics and breeding due to their multi-allelic nature, stability, co-dominant inheritance, and relative abundance in the genome [36,37]. Studies have shown that *Vicia amoena* is a kind of forage grass with high nutritional quality, similar to alfalfa. In the study of the genetic diversity of 24 *V. amoena* individuals, it was found that the range of PIC was from 0.896 to 0.968, with an average of 0.931. This indicates that these markers have a very high information content [38]. Previous studies have suggested that markers with a PIC value greater than 0.5 are highly informative, while those exceeding 0.7 are suitable for genetic mapping [39]. In this study, 49 alfalfa varieties were analyzed using 23 pairs of SSR primers. The results showed that 21 SSR markers exhibited a PIC value greater than 0.5, and 8 markers exceeded 0.7. These SSR markers demonstrate high polymorphism and are appropriate for performing genetic diversity and genetic map analyses. This also indicates that alfalfa possesses abundant genetic differentiation and a relatively complex genetic background.

By analyzing the diversity of various alfalfa populations, we found that the observed number of alleles ranged from 0.941 to 1.581, the diversity index varied from 0.147 to 0.192, and the Shannon index spanned from 0.216 to 0.303 (Table 4). These results indicate a notable level of genetic diversity among the tested populations [40]. An analysis of molecular variance revealed that 2% of the genetic variation exists between populations, while 98% exists within populations, with individual variation being the primary source of total variation (Table 5). Thovhogi et al. found, through an analysis of molecular variance (AMOVA), that the total variation (100%) was entirely derived from intra-individual variation [41]. Wu et al. found that 88% of the variation occurred within populations, and the remaining 12% occurred among populations [38]. Similarly, Su et al. demonstrated using AMOVA that 83% of the genetic variation was within populations, while 17% was among populations [42]. These findings align with the results of this study, reinforcing the conclusion that individual variation is the primary source of total variation.

A cluster analysis of 49 alfalfa varieties was conducted using the UPGMA method based on Nei’s genetic distance. The germplasms from different sources could not be completely distinguished, and the correlation between genetic relationships and geographical distribution was not significant, which aligns with the findings of Wang et al. [43] and Zhou et al. [44]. The cluster analysis results of Touil et al. indicated that the different clusters obtained comprised both local alfalfa populations and cultivated alfalfa populations, suggesting that population clustering is not related to geographical origin. This observation is also consistent with the conclusions drawn from the cluster analysis in this study [35]. However, Shen et al. pointed out that alfalfa varieties from North America and South America were clustered into different groups, which is consistent with the migration and cultivation history of alfalfa. Cultivated alfalfa (*Medicago sativa*) may have originated from an ancestral population in Europe. Alfalfa has continuously enriched its gene pool by absorbing genetic components from other subspecies, enabling it to have a wide distribution and adapt to different environments [45]. Chen et al. re-sequenced 220 core germplasm resources of alfalfa. A population structure analysis showed that alfalfa varieties in China have formed an independent subgroup during the long process of domestication and improvement [46]. Wu et al. found that the special habitats of altitude and geographical origin may be important factors influencing the population clustering patterns of Vicia amoena [38]. The above studies illustrate that the geographical origin plays an important role in the population differentiation of alfalfa. The reasons for this may include the decreasing limitation of parental sources of bred varieties due to the expansion and increased frequency of international circulation of germplasm resources. Additionally, the insufficient number of materials in this study may have contributed to these results, among other factors. Finally, the fingerprint profiles established in this study can serve as a database for alfalfa varieties, providing an excellent tool for the certification and protection of these varieties. However, this study also has limitations: the number of alfalfa materials selected is limited, and future research could benefit from expanding the range of alfalfa materials and increasing both the number and scope of SSR markers to further validate the conclusions drawn here.

## 4. Materials and Methods

### 4.1. Plant Material

A total of 49 alfalfa varieties were used in this experiment. The detailed information on these varieties can be found in Table 6.

### 4.2. Genomic DNA Extraction

The plant genomic DNA extraction kit (Tiangen, Beijing, China) was utilized to extract DNA from the test samples. For each cultivar, tender leaves from 10 independent plants were collected and mixed, followed by DNA extraction. Following extraction, the concentration of the DNA samples was measured using a NanoDrop ONE instrument (ThermoFisher, Waltham, MA, USA). The quality of DNA affected by contaminants such as proteins and phenolic compounds was detected by NanoDrop ONE (260/280 and 260/230 ratios) and the quality of the DNA was assessed via 1.5% agarose gel electrophoresis. Upon evaluation, the DNA samples were stored at −20 °C for future use.

### 4.3. Source and Synthesis of Primers

A total of 102 pairs of primers were selected for testing based on previous studies [26,34]. The primers were synthesized using the adapter method, which involves adding a 21 bp adapter sequence to the upstream primer during synthesis. The primers were commissioned to be synthesized by Sangon Biotech (Shanghai, China) Co., Ltd.

### 4.4. The PCR Reaction Procedure and System

Each reaction system comprises 5 μL of 2× Taq PCR Master Mix, 20 ngof genomic DNA, 0.5 μL each of forward and reverse primers at a concentration of 10 pmol/μL, with deionized distilled water (ddH_2_ O) added to bring the total volume to 10 μL. The reaction steps are as follows: 95 °C pre-denaturation, 5 min; 95 °C denaturation, 30 s; 62~52 °C gradient annealing, 30 s; 72 °C extension, 30 s; 72 °C terminal extension, 20 min, a total of 25 cycles; and the products were stored at 4°. The specific steps for primer screening were as follows: for the first batch of 8 samples, 52 pairs of primers were screened. In the first step, a total of 8 screening samples were randomly selected from the population, and 52 pairs of primers were amplified, resulting in 11 pairs of polymorphic primers. In the second step, the 8 samples were used to re-screen the 11 pairs of primers screened in the previous step, and finally, 8 pairs of polymorphic primers were obtained. For the second batch of 8 samples, 50 pairs of primers were screened. In the first step, the 8 screening samples were used to amplify the 50 pairs of primers in the second batch, and finally, 17 pairs of polymorphic primers were obtained. In the second step, the 8 samples were used to re-screen the 17 pairs of primers screened in the previous step, and finally, 15 pairs of polymorphic primers were obtained. Finally, 23 pairs of polymorphic primers were used to detect 51 samples from the population. All the above reactions were performed on a Veriti384 PCR instrument (Applied Biosystem, Foster City, CA, USA). After the PCR reactions were completed, the amplified products were detected by fluorescence capillary electrophoresis.

### 4.5. Primer Screening

In the primer screening phase, eight samples were selected from the test alfalfa, and 102 pairs of primers were amplified using a Veriti 384 PCR machine (Applied Biosystem, USA). Following the PCR reaction, the amplified products were detected via fluorescent capillary electrophoresis. The results were subsequently analyzed using the GeneMarker software program (3.0.1). The eight samples were then rescreened with the primers identified in the previous step. After the PCR reaction, the amplified products were detected through fluorescent capillary electrophoresis, and the results were analyzed using the GeneMarker software program (3.0.1).

### 4.6. Fluorescence Capillary Electrophoresis Detection

A fragment analysis, separation, and the detection of PCR products were performed on the 3730 Genetic Analyzer (Applied Biosystem, USA). An aliquot (1 μL) of PCR product was added to 9 μL of cocktail, i.e., 8.5 μL Hi-Di™ formamide and 0.5 μL GeneScan™ 500 LIZ. Samples were then denatured for 3 min at 95 °C and immediately chilled on ice for 2 min and loaded on the 3730 Genetic Analyzer (Applied Biosystems, USA) for analysis and the detection of SSR samples.

### 4.7. Construction of DNA Fingerprinting

A total of 23 primer pairs with polymorphism were selected to amplify 49 alfalfa varieties for constructing their fingerprint profiles. Polymorphic bands were visually scored as binary data using GeneRuler™ 100 bp DNA Ladder, with “1” indicating presence and “0” indicating absence. Based on the data matrix, fingerprint codes and QR codes were generated.

### 4.8. Data Analysis

The GeneMarker software program (3.0.1) was used to analyze the results of a fragment analysis achieved by capillary electrophoresis performed at the ABI 3730xL sequencer, and the number of alleles, peak maps, and genotypes of each sample were obtained. The number of alleles (Na), number of effective alleles (Ne), genetic distance, and genetic identity among populations were analyzed using the GenAlEx 6.501 software program. The Excel 2021 software package was used for analyzing and calculating the number of alleles and the polymorphic information content (PICi). Finally, a principal coordinate analysis was performed using the GenAlEx 6.501 software program to study the similarities or differences in the composition of the sample populations.

$$PIC_{i}=1-\sum_{i=1}^{n}P_{i}$$

allele frequency (Pi) $P_{i}=\frac{1}{N}\sum_{i=1}^{n}f_{i}$.

Note: fi represents the amplified band pattern of the i-th DNA sample; N represents the total number of amplified bands.

## 5. Conclusions

The results of this study indicate that SSR markers are effective tools for the genetic characterization and precise discrimination of alfalfa varieties. They can be used to study the genetic traits of 49 alfalfa varieties and have important application values in breeding, variety registration, and germplasm resource conservation. Therefore, the method based on SSR markers provides a reliable and efficient strategy for optimizing the management and commercialization of diverse alfalfa germplasm resources.

## Figures and Tables

**Table 1:** Sequences of 23 primer pairs.

Primer Name	Forward Primer	Reverse Primer
aw01	GAAGGTGACCAAGTTCATGCTACCTGTTCTAAGGGAGATTTCG	CAGGGGAAGCATACAAAACC
AW11	GAAGGTGACCAAGTTCATGCTATTCGCAGTGAGCTGATCCT	GACATTTGCAGACCACCATT
AW166	GAAGGTGACCAAGTTCATGCTAACGTAACGACAGCAACATCA	CAGATTGCATTTTGGGTTCC
aw690665	GAAGGTGACCAAGTTCATGCTGGTTTTGGAGACATGACGGT	GTGAAGACTTTGCGGTGGAT
B14B03	GAAGGTGACCAAGTTCATGCTGCTTGTTCTTCTTCAAGCTCAC	ACCTGACTTGTGTTTTATGC
BI10	GAAGGTGACCAAGTTCATGCTAAAACGGTACCCGTATCAACA	TCTGGAAGATGAGACCGTGA
MTB60	GAAGGTGACCAAGTTCATGCTAAGAATGACGAAGAGGCGAA	TCAGAAATTCCCTCCCATTG
MTIC432	GAAGGTGACCAAGTTCATGCTTGGAATTTGGGATATAGGAAG	GCCATAAGAACTTCCACTT
Act012	GAAGGTGACCAAGTTCATGCTGTTTGTGCAGCCCTTTGATT	ATGCAAACCAAGATTAAGGC
AL73	GAAGGTGACCAAGTTCATGCTGAATAATGCTGGTGGAAGCAA	GTTGAGTTACCCCACATGACAA
AW123	GAAGGTGACCAAGTTCATGCTAGTCCCTGCAAAATCCCTTC	CATGTTTCCGGTTCTGGTTT
AW282	GAAGGTGACCAAGTTCATGCTCGACCAAATCACTCTTCTTCAA	AATCCAAGACCATTCACCTGAG
AW300	GAAGGTGACCAAGTTCATGCTCCACGTTGTGTCATTGTCTACTC	GTCGAAGAAAGAGGTGGTTGTT
aw695900	GAAGGTGACCAAGTTCATGCTGCAACCATCTAAACCCAACAA	AGGCTAATCGACGGGAAAAT
BE112	GAAGGTGACCAAGTTCATGCTTTCATTTCATAGTTTTCCATTGC	AGCGAGATAGATTTCACCGAAG
BE92	GAAGGTGACCAAGTTCATGCTAGTTCAAACCCTTACCCTTCA	GATGAGGATGATGATGAATTGG
BF123	GAAGGTGACCAAGTTCATGCTAGAACCTCGTCATCAGGAACAT	GACAGAGACGGAGAAGGAAGAA
m206	GAAGGTGACCAAGTTCATGCTCCCCATTGACGCATTCTTAC	TCCTCAACCAACCACTTCCT
m230	GAAGGTGACCAAGTTCATGCTTTACCATATTAACCCCCGCA	CGCATATCACCTCCCAGAAT
Ms-27	GAAGGTGACCAAGTTCATGCTGTAGTGAAGGACCAAGAAATGA	CAAGAAATTGTAATCTCCATTG
Ms-64	GAAGGTGACCAAGTTCATGCTCGCTTTCGCTGTCGAACT	GGATTCAGCAACCGGAAA
MT1E04	GAAGGTGACCAAGTTCATGCTTCTAGGTATTCGCTGGCGTT	TGTTTCTGATCAGGGCATTG
MTIC249	GAAGGTGACCAAGTTCATGCTTAGGTCATGGCTATTGCTTC	GTGGGTGAGGATGTGTGTAT

**Table 2:** Genetic diversity analysis.

Name	Number of Alleles	Number of Polymorphic Alleles	Percentage of Polymorphic Loci (%)	Polymorphic Information Content
Act012	4	4	100.00	0.683
AL73	5	4	80.00	0.705
aw01	7	7	100.00	0.748
AW11	6	6	100.00	0.784
AW123	4	4	100.00	0.708
AW166	6	6	100.00	0.698
AW282	3	2	66.67	0.633
AW300	7	6	85.71	0.701
aw690665	5	4	80.00	0.751
aw695900	5	5	100.00	0.347
B14B03	10	10	100.00	0.681
BE112	16	16	100.00	0.804
BE92	6	6	100.00	0.646
BF123	3	3	100.00	0.473
BI10	10	10	100.00	0.674
m206	6	6	100.00	0.561
m230	5	5	100.00	0.694
Ms-27	4	4	100.00	0.689
Ms-64	4	3	75.00	0.558
MT1E04	3	3	100.00	0.649
MTB60	7	6	85.71	0.637
MTIC249	3	2	66.67	0.648
MTIC432	7	7	100.00	0.704
Total	136	129	94.85	15.174
Average	5.91	5.61		0.66

Note: The number of alleles refers to the total number of all alleles present at a specific locus in a population; the number of polymorphic alleles refers to the quantity of alleles with polymorphism at a specific locus in a population; the percentage of polymorphic loci refers to the proportion of polymorphic loci among the total number of tested loci in a population; the polymorphic information content refers to the richness of genetic information that a locus can provide in a population, reflecting the ability of the locus to distinguish between individuals or populations.

**Table 3:** Inter-population genetic diversity.

Pop	Na	Ne	I	H	UH
pop1	1.191 ± 0.078	1.289 ± 0.029	0.267 ± 0.023	0.176 ± 0.016	0.201 ± 0.018
pop2	1.581 ± 0.066	1.311 ± 0.029	0.303 ± 0.021	0.192 ± 0.015	0.2 ± 0.016
pop3	1.265 ± 0.078	1.32 ± 0.03	0.292 ± 0.024	0.192 ± 0.016	0.209 ± 0.018
pop4	0.941 ± 0.077	1.25 ± 0.029	0.216 ± 0.025	0.147 ± 0.017	0.196 ± 0.022
Total	1.244 ± 0.039	1.292 ± 0.015	0.27 ± 0.012	0.177 ± 0.008	0.201 ± 0.009
Average	1.2445	1.2925	0.2695	0.1768	0.202

Note: four populations based on variety origin; Na: observed allele; Ne: effective allele; I: Shannon information index; H: diversity index; UH: unbiased diversity index.

**Table 4:** Molecular analysis of variance (AMOVA) of populations.

Source	df	SS	MS	Est. Var.	%
Among Pops	3	51.340	17.113	0.320	2%
Within Pops	45	618.007	13.733	13.733	98%
Total	48	669.347		14.054	100%

Note: Source: source of variation; df: degrees of freedom; SS: total variance; MS: mean squared error; Est. Var.: estimated value of variance; %: percentage of variation.

**Table 5:** Genetic identity and genetic distance among the populations.

ID	pop1	pop2	pop3	pop4
pop1		0.978	0.956	0.952
pop2	0.023		0.980	0.960
pop3	0.045	0.020		0.949
pop4	0.049	0.041	0.052	

Note: The values above the diagonal represent genetic identity (Gi), and the values below the diagonal represent genetic distance (Gd).

**Table 6:** Alfalfa materials for testing.

Sample	Variety	Origin	Hibernation Level
S1	WL354HQ	America	4
S2	Hetian	China	4
S3	Qianjing	America	-
S4	Jinneng5020	Canada	5
S5	Yongshi	America	-
S7	Longmu803	China	1
S8	Zhongmu No1	China	2
S11	MF4020	Canada	4
S12	Qiji	America	3
S13	Jiguang	Australia	3
S14	Weisheng	America	4.6
S15	Baimu202	America	2
S16	Jianeng	America	4
S17	MT4015	Canada	4
S18	Xunlu	Canada	1
S21	701-421476	-	
S22	42IQ	America	-
S23	Handi	America	3
S25	310SC	America	-
S26	Qingtianzhu	America	4
S27	Qishi No.2	America	3.4
S28	Kangsai	America	3
S29	Longmu 801	China	1
S30	Juneng No.2	America	3.2
S31	4030	Canada	4
S32	Yinhe	America	4.2
S33	6010	China	6
S34	420YQ	America	-
S35	Panshi	America	-
S36	SF8001	Canada	-
S37	Daye No.3	China	-
S38	Kangkuan	-	-
S39	Beijixiong	America	2
S40	Baimu201	America	2
S41	Bolatu	Germany	-
S42	Ningxia xibei	China	-
S43	218TR	America	2
S44	Jinhuanghou	America	2
S45	Yinsite	America	4
S46	Kangsai I	America	3
S47	Xubao	Canada	-
S48	Tiaozhanzhe	America	2.5
S49	Gongnong No.1	China	1
S50	Baimu 341	Canada	3.4
S52	WL168HQ	America	2
S53	Dinamo	-	-
S54	YS401	-	-
S55	YS40	-	-
S56	YS402	-	-

## Data Availability

The original contributions presented in this study are included in the article. Further inquiries can be directed to the corresponding author.
